# Use of the Health Promotion Model by Nursing in Primary Care: an integrative review

**DOI:** 10.1590/0034-7167-2024-0096

**Published:** 2025-03-14

**Authors:** Mayara Góes dos Santos, Nathália Ivulic Pleutim, Andreia Insabralde de Queiroz-Cardoso, Lorraine dos Santos Ramalho, Verusca Soares de Souza, Elen Ferraz Teston

**Affiliations:** I Universidade Federal de Mato Grosso do Sul. Campo Grande, Mato Grosso do Sul, Brasil; II Universidade Estadual do Paraná. Maringá, Paraná, Brasil

**Keywords:** Nursing, Nursing Theory, Primary Health Care, Primary Nursing, Health Promotion, Enfermería, Teoría de Enfermería, Atención Primaria de Salud, Enfermaria Primaría, Promoción de la Salud

## Abstract

**Objective:**

To summarize the scientific literature in nursing that has utilized Nola Pender’s Health Promotion Model in primary care.

**Method:**

An integrative review conducted in March 2023, using databases such as Embase, Elsevier’s Scopus, Biblioteca Virtual em Saúde, Medline via PubMed, Web of Science, and Ovid.

**Results:**

A total of 660 articles were identified, with eight articles included in the final sample. The evidence highlighted the applicability of the Health Promotion Model in various contexts: dietary behaviors in individuals with hypertension, healthy aging, breastfeeding in first-time mothers, and educational programs aimed at improving behavior and increasing knowledge in individuals living with HIV.

**Conclusions:**

The synthesis of the nursing literature that applied Nola Pender’s Health Promotion Model in primary care demonstrated that the model provides a valuable framework for implementing nursing interventions focused on health-promoting behaviors and increasing individuals’ engagement in their own health care.

## RESUMEN

**Objetivo:**

Resumir las producciones científicas de la Enfermería que utilizaron el Modelo de Promoción de la Salud de Nola Pender en la Atención Primaria.

**Método:**

Revisión integrativa, realizada en las bases de datos Embase, Elsevier’s Scopus, Biblioteca Virtual en Salud, Medline vía PubMed, Web of Science y Ovid, en marzo de 2023.

**Resultados:**

Se identificaron 660 artículos, de los cuales ocho compusieron la muestra final. La evidencia destacó la aplicabilidad del Modelo de Promoción de la Salud en diversas condiciones: comportamientos alimentarios de personas con hipertensión; envejecimiento saludable; lactancia materna en primíparas; y en programas de educación para mejorar el comportamiento y aumentar el conocimiento en personas que viven con VIH.

**Conclusiones:**

La síntesis de las producciones científicas de la Enfermería que utilizaron el Modelo de Promoción de la Salud de Nola Pender en la Atención Primaria señaló que dicho modelo proporciona bases para la implementación de intervenciones de enfermería enfocadas en comportamientos promotores de salud y en el compromiso del individuo con su propia salud.

## INTRODUCTION

Health promotion proposes the integration of technical and popular knowledge, the mobilization of resources for quality of life, and shared responsibility for problems and solutions ^(1)^. On a global scale, studies have highlighted the effectiveness of health promotion actions in engaging people in the management of their health conditions ^(2-4)^. In Brazil, the actions developed by the multiprofessional team working in Primary Health Care (PHC) occupy a privileged position due to the frequent interaction between professionals and individuals/families, the established bond, and access to the real needs of the population registered in the designated territory ^(1)^.

Although these teams’ actions include health promotion and protection, disease prevention, diagnosis, treatment, rehabilitation, harm reduction, and health maintenance ^(5)^, it is observed that care efforts are largely concentrated on disease treatment or reducing complications caused by diseases ^(6)^. Studies conducted in Brazil ^(7,8)^ demonstrate the need for a reorientation of the healthcare model so that the focus shifts to the individual and actions promote health-oriented practices. For this, intersectoral actions are needed that consider the influence of social determinants of health on lifestyle habits and recognize personal aspects as influential factors in individual behavior and engagement with their health condition.

Regarding personal factors, the Health Promotion Model (HPM), proposed by Nola Pender in 1980 in the United States, focuses on the influence of self-efficacy (a person’s belief in their ability to achieve a goal), perceived benefits, and barriers to adopting healthy behaviors ^(9)^. The model highlights that each person has unique personal characteristics and experiences that affect their actions, and that the set of variables related to knowledge and specific behavioral effects holds motivational significance, enabling care interventions that support change ^(9,10)^.

Thus, the premises of this model emphasize the need for health promotion actions carried out by nursing to focus on improving the well-being of the individual receiving care. This is because individuals tend to regulate their own behavior, even considering its complexity, allowing them to interact with their environment and transform over time. Health professionals constitute part of the interpersonal environment that influences people throughout their lives, and the self-initiated reconfiguration of person-environment interaction patterns is essential for behavior change and health promotion ^(9,10)^.

Studies ^(11-13)^ that applied Pender’s HPM demonstrated its use, especially among people with health conditions or with a focus on disease prevention actions. In light of the above, the following question arises: how has nursing utilized Nola Pender’s model in health promotion actions developed in PHC? It is expected that the knowledge synthesis produced by this study will assist nurses in identifying biopsychosocial aspects, as proposed by the HPM, that may influence individuals’ engagement in health promotion and self-care actions.

## OBJECTIVE

To summarize the scientific nursing literature that utilized Nola Pender’s HPM in Primary Care.

## METHOD

### Ethical Aspects

The ethical aspects of this study were preserved, and all the authors of the analyzed articles were properly referenced, with their content faithfully presented in accordance with Brazilian Copyright Law 9.610 of 1998.

### Study Design

This is an integrative literature review study, following a rigorous and well-defined method commonly used in evidence-based practice. The steps include problem identification, formulation of the guiding question, literature search with the application of inclusion and exclusion criteria, data collection through a previously structured instrument, data analysis, and presentation of the review ^(14,15)^.

### Formulation of the Research Question

The PVO strategy was used to formulate the guiding question ^(16)^, where P refers to the population of interest or the condition/problem under investigation (Nursing Professionals), V refers to the variable of interest (Nola Pender’s Health Promotion Model), and O, from English outcome, refers to the result/outcome to be analyzed (scientific productions focused on PHC). Thus, the guiding question was defined as: “Which scientific nursing literature has utilized Nola Pender’s HPM in PHC?”

### Literature Search and Sampling

The search was conducted in March 2023 using the proxy of the Federal University of Mato Grosso do Sul to access the CAPES Journal Portal. The electronic databases used were EMBASE, Elsevier’s Scopus (SCOPUS), BVS, National Library of Medicine (Medline via PubMed), Web of Science, and Ovid. The descriptors used were from Medical Subject Headings (MeSH), *Descritores em Ciências da Saúde* (DeCS), and ENTREE, with the application of Boolean operators AND and OR (Chart 1).

**Chart 1:** Search strategies used for the respective databases and the number of findings, 2023

Data base	Search strategies	Results
*Pubmed Central*	*((((Nurses[Title/Abstract]) AND (Health Promotion[Title/Abstract])) OR (Pender’s Model[Title/Abstract])) OR (Pender’s health promotion model[Title/Abstract])) AND (Primary Health Care[Title/Abstract])*	177
*Embase*	*(‘nurse’/exp OR ‘community health nurse’ OR ‘community health nurses’ OR ‘nurse’ OR ‘nurse, community health’ OR ‘nurses’ OR ‘nurses, community health’ OR ‘nurses, public health’ OR ‘nursing assistance’ OR ‘public health nurse’ OR ‘public health nurses’) AND (‘health promotion model’/exp OR ‘health promotion model’ OR ‘Pender health promotion model’ OR ‘Pender’s health promotion model’) AND (‘primary health care’/exp OR ‘first line care’ OR ‘health care, primary’ OR ‘primary care nursing’ OR ‘primary health care’ OR ‘primary healthcare’ OR ‘primary nursing care’)*	6
*Scopus*	***ALL (“Nurses” AND “Health Promotion” AND “Pender health promotion model” AND “Primary Health Care”*)**	32
*BVS*	*A# (nursing) AND (nursing theory) AND (health promotion) AND (primary health care)* *B# (nursing) AND (nursing theory) AND (health promotion) AND (primary health care)*	80 178
*Web of science*	*(((ALL=(Nurses)) AND ALL=(Health Promotion)) AND ALL=(Pender’s health promotion model)) AND ALL=(Primary Health Care)*	2
*Ovid*	*(Nurses and Health Promotion and Primary Health Care).af.*	185
Total		660

Full-text articles were included, with no limitations on language or year of publication. Duplicate articles were excluded, along with editorials, letters to the editor, abstracts, expert opinions, correspondences, reviews, book chapters, theses and dissertations, monographs, and final course papers.

**Figure 1:**
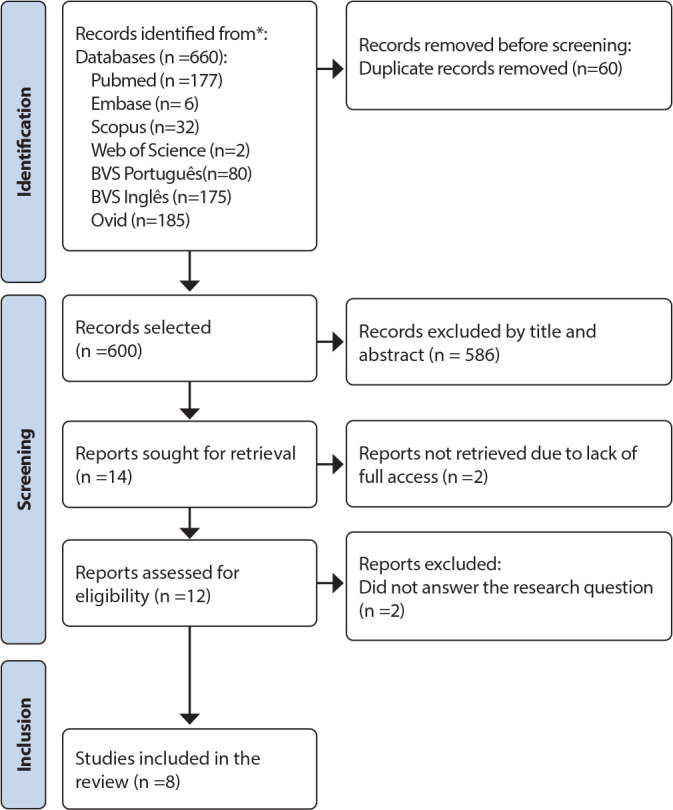
Flowchart of identification, screening, eligibility, and inclusion of studies for the integrative review, 2023

The studies were identified in the selected information sources by two independent researchers, who were previously trained to evaluate titles and abstracts, using a free web-based review program called Rayyan, developed by the Qatar Computing Research Institute ^(17)^. In cases of disagreement, a third researcher was involved in the decision-making process. Following this, the full-text articles were read, and a manual search of the references in the included articles was conducted, although no new additions were made in the end. The selection flowchart is shown in Figure 1.

### Characterization and evaluation of the studies

For data collection, a detailed instrument was used, which included: first author, method, participant characteristics, conclusions, and limitations. Following this, the analysis and synthesis of the articles were conducted descriptively. To evaluate the level of evidence of the studies, the *Rating System for the Hierarchy of Evidence for Intervention/Treatment Questions* was used, which consists of seven levels of evidence: Level I - Systematic review of meta-analyses; Level II - Randomized controlled trials; Level III - Controlled trials without randomization; Level IV - Case-control or cohort studies; Level V - Systematic review of qualitative or descriptive studies; Level VI - Qualitative or descriptive study; Level VII - Expert opinion or consensus ^(18)^.

To systematize the description of the steps in the review process, the guidelines of the Preferred Reporting Items for Systematic Reviews and Meta-Analyses (PRISMA) were followed, adhering to the minimum quality standards for reviews ^(19)^.

## RESULTS

Of these, 60 were removed due to duplication and 586 were excluded after title and abstract screening for not meeting the inclusion criteria. The final sample consisted of 14 articles, with four excluded due to lack of full-text access, and after reading the full texts, two articles were excluded for not addressing the guiding question. Therefore, in this integrative review, eight articles were analyzed.Nola Pender’s HPM in PHC was used in studies to analyze the promotion of healthy aging ^(20,21)^, support for breastfeeding and maternal self-efficacy ^(22,23)^, healthy lifestyle and disease prevention among workers ^(24,25)^, perceptions of barriers and learning for people living with HIV/AIDS ^(26)^, and improvements in health-promoting behaviors ^(27)^ (Chart 2).

**Chart 2:** Scientific Nursing Productions that used Nola Pender’s HPM in Primary Health Care, 2023

Authorship, year of publication, and country	Objective	Type of study/sample and data collection instrument	Conclusions	Level of evidence
Abbas, 2022^(25)^ Iraq	To identify the effectiveness of Pender’s HPM in interventions aimed at improving the dietary behaviors of hypertensive employees.	Randomized clinical trial conducted with 220 employees from a university in Mosul, diagnosed with hypertension. The intervention was based on Pender’s HPM premises and focused on dietary habits.	The intervention based on Pender’s HPM had a positive impact on the perceived benefits of following a healthy diet, the risks of not following it, self-efficacy, and social support to assist with the change.	II
Cardoso, 2021^(20)^ Brazil	To model the process of promoting healthy aging based on the conceptual analysis of Walker and Avant and Nola Pender’s Health Promotion Model.	Integrative review conducted with 36 studies from the databases: CINAHL, PubMED (including MEDLINE), Scopus, and Web of Science, using the descriptor “Healthy Aging.”	HPM can guide the application of the nursing process with a focus on healthy aging.	VI
Rababa, 2021^(21)^ Jordan	To examine health-promoting behaviors, health needs, and associated factors among the elderly in Jordan.	Cross-sectional study conducted with 220 elderly individuals attending outpatient clinics at two hospitals. A scale (Health-Promoting Lifestyle Profile) based on Pender’s HPM was applied.	Despite the overall good average scores of the elderly on the total Health-Promoting Lifestyle Profile and in the dimensions of self-actualization, health responsibility, and interpersonal relationships, the level of exercise and physical activity was poor.	IV
Necipoÿlu, 2021^(23)^ Cyprus	Determine the effect of nursing interventions based on Dennis’s Breastfeeding Self-Efficacy Theory and Pender’s Health Promotion Model.	Randomized controlled study conducted with 60 first-time immigrant mothers, divided into two groups (intervention and control). For the intervention, a Breastfeeding Training Guidebook and the guidelines from Dennis’s Breastfeeding Self-Efficacy Theory and Pender’s HPM were used. Data were collected using the Breastfeeding Self-Efficacy Scale and the Breastfeeding Assessment Tool.	There was a statistically significant difference between the scores of the Breastfeeding Self-Efficacy Scale, the Breastfeeding Assessment Tool, and Breastfeeding Self-Efficacy between the groups. The breastfeeding self-efficacy score of the experimental group increased more than expected.	II
Sari, 2020^(22)^ Turkey	Examine the effectiveness of a web-based program, based on Nola Pender’s Health Promotion Model, for first-time mothers in breastfeeding.	Randomized controlled trial conducted with 71 first-time pregnant women (35 in the intervention group and 35 in the control group). The research was carried out in four stages: creation of professional content for the web-based educational program; design and pre-application of the content; implementation and evaluation of the results. The topics and subjects were pre-compared within Pender’s framework.	Women who participated in the web-based program demonstrated better self-efficacy, and their babies showed better scores in measures of growth, development, and health compared to those in the control group.	II
Heydari, 2014^(27)^ Pakistan	Identify the effects of the intervention program in improving health-promoting behaviors.	Narrative review with a descriptive synthesis of 74 articles. The search was conducted using the following databases: PubMed, Google Scholar, Proquest, Elsevier. The search terms used were: Pender, Health, Health Promotion, and Health Promotion Model.	The behavioral factors identified that showed improvement after the interventions were: self-actualization, health responsibility, physical exercise, nutrition, interpersonal support, and stress management.	VI
Eshah, 2010^(24)^ Jordan	Evaluate the effectiveness of an education, counseling, and behavioral skill development program for Jordanian working adults to adopt a healthy lifestyle, based on Pender’s Health Promotion Model.	Quasi-experimental study conducted with 123 adults, aged between 22 and 60 years, who worked in schools and were allocated by school to form the experimental and control groups. The experimental group received group education and individual counseling, in addition to techniques for developing behavioral skills. Pender’s HPM was used to formulate the strategy for counseling and developmental interventions. Data were collected using a questionnaire measuring knowledge, attitude, and belief, as well as the Health Promotion Lifestyle Profile II questionnaire.	The results confirm the importance of interventional programs in improving participants’ knowledge and attitudes towards coronary artery disease.	III
Mendias, 2007^(26)^ United States of America	Examine perceptions regarding learning needs and interests related to self-care, barriers, and preferred learning modalities, based on Nola Pender’s Health Promotion Model.	Exploratory study conducted with 151 individuals with Human Immunodeficiency Virus, actively receiving outpatient care, aged 18 years or older. Interviews were conducted using questions that addressed perceived learning needs about health and self-care, as well as barriers and preferred learning modalities for outpatient HIV/AIDS patients.	Most expressed interest in health and self-care. Many identified various topics as learning barriers and preferred learning modalities.	IV

## DISCUSSION

The studies analyzed in this review indicated the use of Pender’s HPM by nurses in caring for individuals across different life stages and health conditions ^(20-27)^.

In the analysis of the evidence hierarchy levels ^(18)^, three studies were classified as Level II ^(22,23,25)^, one as Level III ^(24)^, two as Level IV ^(21,26)^, and two as Level VI (20,27), demonstrating the development of studies aimed at generating high-quality and robust scientific evidence. This is essential and indispensable for supporting nurses’ decision-making and improving the quality of care provided.

Regarding the applicability of Nola Pender’s theories in the studies analyzed, it was observed that they were employed both for the formulation of health interventions ^(20,22-25)^ and for the analysis of health-promoting behaviors and perceptions ^(21,26,27)^.

### Formulation of Health Interventions

One of the intervention studies analyzed in this review indicated that an intervention conducted through home visits for health education, based on Pender’s HPM, facilitated the identification of behaviors, self-efficacy, and personal factors (physical, social, and psychological) that impact an individual’s engagement in self-care activities. It was found that a mother’s positive perception of self-efficacy regarding breastfeeding was associated with the establishment of breastfeeding (duration, the effort required to dedicate time and care for her baby, thoughts about breastfeeding, and the ability to handle difficulties) ^(23)^.

In this context, it is essential to emphasize the need for teams working in PHC to continuously revisit the fundamental principles of territorialization and use home visits as an opportunity to get to know the families registered in the area and identify health needs to direct strategic health promotion actions that address the different realities of each territory. Furthermore, strengthening the bond between the nurse and the service user provides the professional with the opportunity to assist in the process of reflecting on life habits and adopted behaviors, as well as identifying barriers, advantages, and self-efficacy related to behavior changes that impact health promotion, in accordance with the model being discussed.

A randomized controlled clinical trial based on the HPM used an intervention tool in the form of a website with educational content, videos, and instruments that featured a question menu for mothers to ask questions related to breastfeeding.

It was observed that by the third month of follow-up, 88.6% of the babies in the intervention group were breastfeeding, compared to 33.3% in the control group. Regarding the premature introduction of food, 11.4% of the babies in the intervention group were affected, compared to 66.7% of infants in the control group ^(22)^. In light of this, within the PHC context, the relevance and necessity of follow-up actions performed by Family Health teams concerning the effectiveness of health promotion actions stand out.

Considering pregnancy and the postpartum period, for instance, as a phase in the life cycle for some women, the bond established and the continuous care and follow-up actions adopted by the health team, within the context of PHC, can help women recognize the professionals as part of the social support network, which is crucial for adopting healthy habits and behaviors.

In turn, an intervention study composed of counseling sessions and behavioral skill development, based on Pender’s HPM, indicated that the experimental group showed better scores regarding responsibility for their own health, nutrition, and interpersonal relationships ^(24)^. The fact that HPM prioritizes participants’ self-perception concerning their behaviors, fostering the development of new habits and improving existing ones, can contribute to individuals’ engagement with their own health.

While discussions about the prevention of complications in people with chronic diseases are common, addressing health promotion actions (which incorporate the impact of actions on quality of life) is also a necessary element for comprehensive care in the PHC context. Related to this, a study that used HPM as the basis for an intervention conducted with individuals with hypertension showed improvements in the clinical profile and self-efficacy perceptions of the participants in this group ^(25)^.

In addition to the intervention studies analyzed, an integrative review described a theoretical model developed from the application of the healthy aging concept within the framework of Pender’s HPM, forming a structure capable of guiding the operationalization of the nursing process focused on promoting healthy aging ^(20)^. This result can provide a foundation for discussing the actions developed in PHC with a focus on promoting the health of the elderly population.

### Analysis of Health-Promoting Behaviors and Perceptions

The use of HPM for analyzing health-promoting behaviors in the elderly ^(21)^ and identifying perceptions about learning and self-care in people living with HIV ^(26)^ highlighted the relevance of this model for professionals in understanding the dimensions of health-promoting behaviors, as well as the inclusion of individual perceptions to engage users in self-care activities. Although health conditions and illnesses are also influenced by social determinants, which are not always subject to individual intervention, using Pender’s HPM to design health promotion actions allows for the identification of personal aspects related to beliefs and motivation for behavior change, which can reflect in self-care.

Health promotion actions create a space for implementing health education that enables individuals to make informed decisions (considering their specific reality) and reflect on their current behaviors. Becoming aware of habits and behaviors increases the likelihood that individuals will engage with their health condition and be ready for change ^(27)^. In this regard, it is important to note that the biomedical care model, which focuses on disease and medication prescriptions, does not address the complexity of factors influencing health conditions and illnesses in the population, reinforcing the need to incorporate care technologies centered on the individual and their specificities.

A study aimed at analyzing the nursing approach based on Pender’s HPM in the care of individuals with altered sleep patterns identified that the use of electronic devices was the main barrier to behavior change aimed at promoting sleep. Based on this result, an intervention was proposed to reduce the use of cell phones and televisions, implement relaxation techniques, and establish a set bedtime. After the intervention, a positive impact on participants’ sleep patterns was observed ^(28)^.

The importance of health professionals developing actions that encourage and support users’ self-care processes, taking into account their individual needs and preferences, is reiterated. Self-care is a practice carried out by the individual for themselves, developed authentically by reviewing values and principles through self-reflection, demonstrating care behaviors, and considering the entirety of their bodily dimensions (physical, mental, and spiritual), which are reflected in their environment ^(29)^. According to HPM principles, it is emphasized that the central objective of planned care actions must be linked to a behavior that the individual can intervene upon, as the more confident they feel that the goal is achievable, the greater their self-confidence and self-efficacy ^(9)^.

Even when individuals are placed at the center of care and their preferences are respected, they may initially have difficulty setting goals, and this is where the nurse acts as a “supporter” in the process of self-evaluating their behaviors, health condition, and in the development of a care plan. Providing options allows individuals to choose and increase personal control over the proposed behavior.

### Study Limitations

It should be noted that only one of the articles analyzed used all the categories of HPM. However, this does not invalidate the results found, as the model allows categories to be addressed separately, and depending on the result the author seeks to achieve, there may be no need to use all the categories.

### Contributions to Nursing and Public Health

The results of this study clarified the applicability of Pender’s HPM in various contexts. The planning of health promotion actions by nurses in PHC must consider the biopsychosocial aspects of the individual to foster their engagement in managing their own health.

## CONCLUSION

The scientific nursing literature that used Nola Pender’s HPM in PHC highlighted its positive impact on the perception of self-efficacy, recognition of advantages and barriers related to behavior change, increased individual engagement with their health condition, and the role of social support.

However, it is important to note that these results are not sufficient to affirm the model’s effectiveness in the nursing workflow in PHC, given the complexity and multidetermination of health and illness conditions. This is because the studies measured the positive impact of using the model on behavior change in various ways, including self-administered questionnaires, interviews, and specific health indicators, which may limit the comparability of the results. Furthermore, the influence of social determinants of health on individuals’ habits and behaviors, which are factors requiring intersectoral interventions, cannot be disregarded.

## References

[1] Buss MP, Hartz AMZ, Pinto FL, Rocha MF. (2020). Health promotion and quality of life: a historical perspective of the last two 40 years (1980-2020). Ciên Saúde Coletiva.

[2] Santis KK, Mergenthal L, Christianson L, Zeeb H. (2022). Digital technologies for health promotion and disease prevention in older people: protocol for a scoping review. JMIR Res Protoc.

[3] Félix A, Días S. (2023). Digital communication: strategy in health literacy. Rev Cienc Comum Inf.

[4] Araújo MH, Daher VD, Brito SI, Teixeira ER, Morais CH, Pinto AA (2023). Preced-Proceed model as a tool for the construction and evaluation of interventions in workers’ health: methodological study. Contrib Cienc Soc.

[5] Organização Panamericana de Saúde (OPAS) (2023). Five-year report 2018-2022 of the director of the Pan-American Sanitary Department.

[6] Facchini L, Thomasi E, Dilélio A. (2018). Quality of primary health care in Brazil: advances, challenges and perspectives. Saúde Debate.

[7] Souza LE, Paim JS, Teixeira CF, Bahia L, Guimarães R, Almeida N (2019). The current context of health policy and systems research. Ciênc Saúde Coletiva.

[8] Telo MA. (2019). Literacia em saúde na prática. Instituto Universitário de Ciências Psicológicas, Sociais e da Vida Repository.

[9] Pender NJ, Murdaugh CL, Parsons MA. (2014). Health promotion in nursing practice.

[10] Santi DB, Baldissera VDA, Pender NJ, Murdaugh CL, Parsons MA. (2023). Health promotion in nursing practice. Saúde Debate.

[11] Oliveira JLT. (2021). Assistência de enfermagem fundamentada pelo modelo de Nola Pender na prevenção do câncer cervical. Rev Enferm UFJF.

[12] Oliveira SG. (2021). Promoção da saúde de idosas com osteoporose: uma abordagem a partir do modelo de promoção da saúde de Nola Pender.

[13] Ribeiro WA, Neves KC, Fassarella BPA, Souza JGM, Santos LCA, Guedes MMF (2023). Contributos das teorias de Nola Pender e Dorothea Orem para a qualidade de vida da criança com transtorno do espectro autista. CLCS.

[14] Whittemore R, Knafl K. (2005). The integrative review: updated methodology. J Adv Nurs.

[15] Stillwen S, Fineout-Overholt E, Melnyk B, Williamson K. (2010). Evidence-based practice, step by step: searching for the evidence. Am J Nurs.

[16] Biruel E, Pinto R. (2011). In: XXIV Congresso Brasileiro de Biblioteconomia, Documentação e Ciência da Informação.

[17] Ouzzani M, Hammady H, Fedorowicz Z (2016). Rayyan: a web and mobile app for systematic reviews. Syst Rev.

[18] Melnyk BM, Fineout E. (2015). Evidence-based practice in nursing & healthcare: a guide to best practice.

[19] Page MJ, Mackenzie JE, Bossuyt MP. (2022). The PRISMA 2020 statement: updated guidance for reporting systematic reviews. Epidemiol Serv Saúde.

[20] Cardo RB, Caldas CP, Brandão MA. (2022). Healthy aging promotion model referenced in Nola Pender theory. Rev Bras Enferm.

[21] Rababa M, Ali M, Alshaman A. (2021). Health-promoting behaviors, health needs and associated factors among older adults in Jordan: a cross-sectional study. Int J Community Based Nurs Midwifery.

[22] Sari C, Altay N. (2020). Effects of providing nursing care with a web-based program on maternal self-efficacy and infant health. Public Health Nurs.

[23] Necipoglu D, Bebiş H, Seviğ Ü. (2021). The effect of nursing interventions on immigrant women living in Northern Cyprus on their breastfeeding self-efficacy and success: a randomized controlled trial. Health Care Women Int.

[24] Eshah NF, Bond AE, Froelicher ES. (2010). The effects of a cardiovascular disease prevention program on knowledge and adoption of a heart-healthy lifestyle in Jordanian working adults. Eur J Cardiovasc Nurs.

[25] Abbas A, Younis N. (2022). Efficacy of Pender’s health promotion-based model on intervention for enhancing University of Mosul hypertensive employees’ eating behaviors: a randomized controlled trial. Bionatura.

[26] Mendias EP, Paar DP. (2007). Perceptions of health and self-care learning needs of outpatients with HIV/AIDS. J Community Health Nurs.

[27] Heydari A, Khorashadizadeh F. (2014). Pender’s health promotion model in medical research.

[28] Guevara HC (2021). E. Nursing approach based on the Nola Pender model of sleep habits. Rev Ene Enferm.

[29] Saldanha X, Santos I, Silva FVC. (2017). Promoting self-care in clients on hemodialysis: application of the Nola Pender’s diagram. Rev Pesqui Cuid Fundam.

