# Supplementary material for: Use of the Health Promotion Model by Nursing in Primary Care: an integrative review
Source: Rev Bras Enferm. 2025 Mar 14;78(2):e20240096. doi: 10.1590/0034-7167-2024-0096 (PMC11913135; doi:10.1590/0034-7167-2024-0096)
Supplement: Translation

# Translation: Utilização do Modelo de Promoção da Saúde pela Enfermagem na Atenção Primária: revisão integrativa

**DOI:** 10.1590/0034-7167-2024-0096pt

**Authors:** Mayara Góes dos Santos, Nathália Ivulic Pleutim, Andreia Insabralde de Queiroz-Cardoso, Lorraine dos Santos Ramalho, Verusca Soares de Souza, Elen Ferraz Teston

**Affiliations:** I Universidade Federal de Mato Grosso do Sul. Campo Grande, Mato Grosso do Sul, Brasil; II Universidade Estadual do Paraná. Maringá, Paraná, Brasil

**Keywords:** Enfermagem, Teoria de Enfermagem, Atenção Primária à, Saúde, Enfermagem Primária, Promoção da Saúde

## Abstract

**Objetivo:**

Sumarizar as produções científicas da Enfermagem que utilizaram o Modelo de Promoção da Saúde de Nola Pender na Atenção Primária.

**Método:**

Revisão integrativa, realizada nas bases de dados Embase, Elsevier’s Scopus, Biblioteca Virtual em Saúde, Medline via PubMed, Web of Science e Ovid, em março de 2023.

**Resultados:**

Foram identificados 660 artigos, dos quais oito compuseram a amostra final. As evidências destacaram a aplicabilidade do Modelo de Promoção da Saúde em diversas condições: comportamentos alimentares de pessoas com hipertensão; envelhecimento saudável; aleitamento materno em primíparas; e em programas de educação para melhora do comportamento e aumento do conhecimento em pessoas vivendo com HIV.

**Conclusões:**

A síntese das produções científicas da Enfermagem que utilizaram o Modelo de Promoção da Saúde de Nola Pender na Atenção Primária apontou que o referido modelo oferece subsídios para a implementação de intervenções de enfermagem com enfoque em comportamentos promotores de saúde e no engajamento do indivíduo com sua própria saúde.

## INTRODUÇÃO

A promoção da saúde propõe a articulação de saberes técnicos e populares, a mobilização de recursos para a qualidade de vida e a responsabilidade múltipla por problemas e soluções ^(1)^. No contexto mundial, estudos apontaram a efetividade de ações de promoção da saúde no engajamento das pessoas no cuidado com sua condição de saúde ^(2-4)^. No Brasil, as ações desenvolvidas pela equipe multiprofissional atuante na Atenção Primária à Saúde (APS) ocupam um espaço privilegiado, devido à interação frequente entre os profissionais e os indivíduos/famílias, ao vínculo estabelecido e ao acesso às reais necessidades da população cadastrada no território adstrito ^(1)^.

Embora as ações desenvolvidas por essas equipes contemplem a promoção e proteção da saúde, a prevenção de agravos, o diagnóstico, o tratamento, a reabilitação, a redução de danos e a manutenção da saúde ^(5)^, verifica-se que as ações de cuidado estão majoritariamente concentradas no tratamento de doenças ou na redução de complicações por elas causadas ^(6)^. Estudos realizados no Brasil ^(7,8)^ demonstram a necessidade de reorientação do modelo assistencial para que o foco esteja no indivíduo e que as ações oportunizem práticas de promoção da saúde. Para isso, são necessárias ações intersetoriais, que considerem a influência dos determinantes sociais da saúde nos hábitos de vida e a compreensão de aspectos pessoais como influentes no comportamento e no engajamento dos indivíduos com sua condição de saúde.

No que tange aos aspectos pessoais, o Modelo de Promoção da Saúde (MPS), proposto por Nola Pender em 1980 nos Estados Unidos, tem como enfoque a influência da autoeficácia (crença de uma pessoa em sua capacidade de atingir um objetivo), dos benefícios e das barreiras percebidas para a adoção de comportamentos saudáveis^(9)^. O modelo destaca que cada pessoa tem características pessoais e experiências únicas que afetam suas ações e que o conjunto de variáveis para conhecimento e efeito específicos comportamentais tem significado motivacional, o que possibilita intervenções de cuidado que favoreçam a mudança ^(9,10)^.

Assim, as premissas do referido modelo direcionam para a necessidade de que as ações de promoção da saúde realizadas pela enfermagem sejam voltadas para aumentar o nível de bem-estar do indivíduo que recebe o cuidado. Isso porque o fato de os indivíduos procurarem regular seu próprio comportamento, mesmo considerando a complexidade, permite que eles interajam com o ambiente e sejam transformados ao longo do tempo. Os profissionais de saúde constituem uma parte do ambiente interpessoal que exerce influência sobre as pessoas ao longo de sua vida, e a reconfiguração autoiniciada dos padrões interativos pessoa-ambiente é essencial para a mudança de comportamento e a promoção da saúde ^(9,10)^.

Estudos ^(11-13)^ que utilizaram o MPS de Pender apontaram sua aplicação especialmente em pessoas com agravos de saúde ou com foco em ações de prevenção de doenças. Diante do exposto, questiona-se: como a Enfermagem tem utilizado o modelo de Nola Pender nas ações de promoção da saúde desenvolvidas na APS? Espera-se que a síntese do conhecimento produzida pelo presente estudo possa auxiliar os enfermeiros a identificarem aspectos biopsicossociais, conforme proposto pelo MPS, que podem influenciar no engajamento dos indivíduos com as ações de promoção da saúde e de autocuidado.

## OBJETIVO

Sumarizar as produções científicas da Enfermagem que utilizaram o MPS de Nola Pender na APS.

## MÉTODO

### Aspectos Éticos

Os aspectos éticos deste estudo foram preservados, e todos os autores dos artigos analisados foram devidamente referenciados, tendo seu conteúdo apresentado de forma fidedigna, conforme a Lei de Direitos Autorais 9.610 de 1998.

### Delineamento da Pesquisa

Trata-se de um estudo de revisão integrativa de literatura, o qual consiste em um método rigoroso, definido e utilizado pela prática baseada em evidências. Foram seguidos os passos de identificação do problema e elaboração da questão norteadora; busca na literatura com aplicação de critérios de inclusão e exclusão; coleta de dados por meio de instrumento previamente estruturado; análise de dados e apresentação da revisão ^(14,15)^.

### Elaboração da questão de pesquisa

Para a elaboração da questão norteadora, foi utilizada a estratégia PVO ^(16)^, na qual a letra P se refere à população de interesse ou à condição/problema investigado (Profissionais de Enfermagem); a letra V trata da variável de interesse (Modelo de Promoção da Saúde de Nola Pender); e a letra O, do inglês *outcome*, remete ao resultado/desfecho a ser analisado (Produções científicas com enfoque na APS). Desta forma, foi definida a questão norteadora: “Quais produções científicas da Enfermagem utilizaram o MPS de Nola Pender na APS?”

### Busca na literatura e amostragem

A busca ocorreu no período de março de 2023, utilizando o Proxy da Universidade Federal do Mato Grosso do Sul para acesso ao Portal de Periódicos CAPES. As bases de dados eletrônicas utilizadas foram EMBASE, Elsevier’s Scopus (SCOPUS), BVS, National Library of Medicine (Medline via PubMed), Web of Science e Ovid. Os descritores utilizados foram do *Medical Subject Headings* (MeSH), Descritores em Ciências da Saúde (DeCS) e ENTREE, com aplicação dos operadores booleanos AND e OR (Quadro 1).

**Quadro 1:** Estratégias de busca utilizadas para as respectivas bases de dados e número de achados, 2023

Base de dados	Estratégia de busca	Resultados
*Pubmed Central*	*((((Nurses[Title/Abstract]) AND (Health Promotion[Title/Abstract])) OR (Pender’s Model[Title/Abstract])) OR (Pender’s health promotion model[Title/Abstract])) AND (Primary Health Care[Title/Abstract])*	177
*Embase*	*(‘nurse’/exp OR ‘community health nurse’ OR ‘community health nurses’ OR ‘nurse’ OR ‘nurse, community health’ OR ‘nurses’ OR ‘nurses, community health’ OR ‘nurses, public health’ OR ‘nursing assistance’ OR ‘public health nurse’ OR ‘public health nurses’) AND (‘health promotion model’/exp OR ‘health promotion model’ OR ‘Pender health promotion model’ OR ‘Pender’s health promotion model’) AND (‘primary health care’/exp OR ‘first line care’ OR ‘health care, primary’ OR ‘primary care nursing’ OR ‘primary health care’ OR ‘primary healthcare’ OR ‘primary nursing care’)*	6
*Scopus*	*ALL (“Nurses” AND “Health Promotion” AND “Pender health promotion model” AND “Primary Health Care”*)	32
*BVS*	*A# (nursing) AND (nursing theory) AND (health promotion) AND (primary health care)* *B# (nursing) AND (nursing theory) AND (health promotion) AND (primary health care)*	80 178
*Web of science*	*(((ALL=(Nurses)) AND ALL=(Health Promotion)) AND ALL=(Pender’s health promotion model)) AND ALL=(Primary Health Care)*	2
*Ovid*	*(Nurses and Health Promotion and Primary Health Care).af.*	185
Total		660

Foram incluídos artigos disponíveis na íntegra, sem limitação de idioma ou ano de publicação. Por sua vez, foram excluídos os artigos duplicados, além de editoriais, cartas ao editor, resumos, opiniões de especialistas, correspondências, resenhas, capítulos de livros, teses e dissertações, monografias e trabalhos de conclusão de curso.

Os estudos foram identificados nas fontes de informação selecionadas por dois pesquisadores independentes, previamente treinados para avaliar títulos e resumos, por meio de um programa de revisão gratuito da web, de versão única, chamado *Rayyan Qatar Computing Research Institute* ^(17)^. Na ocorrência de divergências, um terceiro pesquisador participou da análise para a tomada de decisão. Em seguida, foi realizada a leitura dos artigos na íntegra e a busca manual das referências dos artigos incluídos, sem resultar, porém, em novos acréscimos ao final. O fluxograma de seleção foi organizado na Figura 1.

**Figura 1:** Fluxograma de identificação, triagem, elegibilidade e inclusão dos estudos de revisão integrativa, 2023

### Caracterização e avaliação dos estudos

Na coleta dos dados, utilizou-se um instrumento detalhado contendo: primeiro autor, método, caracterização dos participantes, conclusão e limitação. Após isso, realizaram-se a análise e a síntese dos artigos obtidos de forma descritiva. Para a avaliação do nível de evidência dos estudos, foi utilizada a escala *Rating System for the Hierarchy of Evidence for Intervention/Treatment Questions*, a qual contém sete níveis de evidência: Nível I - Revisão sistemática de meta-análises; Nível II - Ensaios controlados randomizados; Nível III - Ensaios controlados sem randomização; Nível IV - Estudo de caso-controle ou coorte; Nível V - Revisão sistemática de estudos qualitativos ou descritivos; Nível VI - Estudo qualitativo ou descritivo; Nível VII - Opinião ou consenso de especialistas ^(18)^. Para sistematizar a descrição das etapas de elaboração da revisão, seguiu-se a declaração dos *Principais Itens para Relatar Revisões Sistemáticas e Meta-análises* (PRISMA), atendendo-se aos padrões mínimos de qualidade de revisões ^(19)^.

## RESULTADOS

Destes, 60 foram removidos por duplicidade e 586 excluídos após a leitura de título e resumo, por não se enquadrarem nos critérios de inclusão. A amostra final foi composta por 14 artigos, sendo que quatro foram excluídos devido à indisponibilidade de acesso na íntegra e, após a leitura dos textos completos, dois artigos foram excluídos por não responderem à pergunta norteadora. Portanto, nesta revisão integrativa, foram analisados oito artigos.

O MPS de Nola Pender, na APS, foi utilizado em estudos para análise da promoção do envelhecimento saudável ^(20,21)^, apoio ao aleitamento materno e autoeficácia materna ^(22,23)^, estilo de vida saudável e prevenção de agravos em trabalhadores ^(24,25)^, percepção de barreiras e aprendizagem para pessoas convivendo com HIV/AIDS ^(26)^ e melhorias de comportamentos promotores de saúde ^(27)^ (Quadro 2).

**Quadro 2:** Produções Científicas da Enfermagem que utilizaram o Modelo de Promoção da Saúde de Nola Pender na Atenção Primária à Saúde, 2023

Autoria, ano de publicação e país	Objetivo	Tipo de estudo/amostra e instrumento de coleta de dados	Conclusões	Nível de evidência
Abbas, 2022^(25)^ Iraque	Identificar a eficácia do modelo baseado na promoção da saúde de pender na intervenção para melhorar os comportamentos alimentares dos funcionários hipertensos.	Ensaio clínico randomizado, realizado com 220 funcionários, de uma universidade de Mosul, com diagnóstico de hipertensão. A intervenção foi realizada com base nas premissas do MPS de Pender e abordou os hábitos alimentares.	A intervenção baseada no MPS de Pender apresentou impacto positivo nos benefícios percebidos de seguir uma dieta saudável, os riscos de não seguir, a autoeficácia e o apoio social para auxiliar a mudança.	II
Cardoso, 2021^(20)^ Brasil	Modelar o processo de promoção do envelhecimento saudável a partir da análise conceitual de Walker e Avant e do MPS de Nola Pender.	Revisão integrativa realizada a partir de 36 estudos, nas bases de dados:CINAHL, PubMED, que inclui a MEDLINE, Scopus e Web of Science, com o descritor “*Healthy Aging*”.	O MPS pode orientar a aplicação do processo de enfermagem com enfoque no envelhecimento saudável.	VI
Rababa, 2021^(21)^ Jordânia	Examinar comportamentos promotores de saúde, necessidades de saúde e fatores associados entre idosos na Jordânia.	Estudo transversal realizado com 220 idosos que frequentavam os ambulatórios dos dois hospitais. Foi aplicada uma escala *(Health-Promoting Lifestyle Profile*) baseada no MPS de Pender.	Apesar da boa pontuação média geral dos idosos no *Health-Promoting Lifestyle Profile* total e nas dimensões autorrealização, responsabilidade com a saúde e relações interpessoais, o nível de exercício e atividade física foi ruim.	IV
Necipoÿlu, 2021^(23)^ Chipre	Determinar o efeito das intervenções de enfermagem baseadas na Teoria de Autoeficácia em Amamentação de Dennis e no MPS de Pender.	Estudo randomizado controlado realizado com 60 mulheres imigrantes primíparas, divididas em dois grupos (intervenção e controle). Para intervenção utilizou-se de uma Cartilha Guia de Treinamento em Amamentação e das diretrizes da Teoria da Autoeficácia na Amamentação de Dennis e do MPS de Pender. Os dados formam coletados por meio da Escala de Autoeficácia na Amamentação e a Ferramenta de Avaliação da Amamentação.	Houve diferença estatisticamente significativa entre os escores da Escala de Autoeficácia em Amamentação e Ferramenta de Avaliação de Amamentação e a Autoeficácia na Amamentação entre os grupos. O escore de autoeficácia em amamentar do grupo experimental aumentou mais que o esperado.	II
Sari, 2020^(22)^ Turquia	Examinar a eficácia de um programa web, baseado no MPS de Nola Pender, para mulheres primíparas no aleitamento.	Ensaio controlado randomizado realizado com 71 gestantes primíparas (35 grupos intervenção e 35 grupos controles). A pesquisa foi realizada em quatro etapas: a criação de conteúdo profissional para o programa educacional baseado na web; projeto e pré aplicação do conteúdo; implementação e avaliação dos resultados. Os temas e assuntos foram pré comparados dentro da estrutura de Pender.	As mulheres que participaram do programa baseado na web demonstraram melhor autoeficácia e seus bebês demonstraram melhores pontuações em medidas de crescimento, desenvolvimento e saúde do que aqueles no grupo de controle	II
Heydari, 2014^(27)^ Paquistão	Detectar efeitos do programa de intervenção para melhorar os comportamentos de promoção da saúde.	Revisão narrativa com síntese descrita de 74 artigos. A busca foi realizada utilizando as bases de dados: Pub Med, Google Scholar, Proquest, Elsevier. Os termos de busca utilizados foram: Pender, Saúde, Promoção da saúde e MPS.	Os fatores comportamentais identificados que apresentaram mudança após as intervenções foram: autorrealização, responsabilidade pela saúde, exercício físico, nutrição, apoio interpessoal e gestão do stress.	VI
Eshah, 2010^(24)^ Jordânia	Avaliar a eficácia de um programa de educação, aconselhamento e desenvolvimento de competências comportamentais em adultos trabalhadores jordanianos para adoção de um estilo de vida saudável, baseado no MPS de Nola Pender.	Estudo quase experimental realizado com 123 adultos, entre 22 e 60 anos, trabalhadores de escolas, os quais foram alocados por escola para compor grupo experimental e grupo controle. O grupo experimental recebeu educação em grupo e aconselhamento individual, além de técnicas de desenvolvimento de habilidades comportamentais. O MPS de Pender foi utilizado para formular a estratégia para o aconselhamento e intervenções de desenvolvimento. Utilizou-se para coleta de dados: um questionário que mede o conhecimento, atitude e crença, e o questionário Perfil de Estilo de Vida e Promoção da Saúde II.	Os resultados confirmam a importância de programas interventivos no conhecimento e atitude dos sujeitos em relação à doença coronariana.	III
Mendias, 2007^(26)^ Estados Unidos da América	Examinar as percepções sobre as necessidades e interesses de aprendizagem de autocuidado, barreiras e modalidades de aprendizagem preferidas, baseado no MPS de Nola Pender.	Estudo exploratório realizado com 151 pessoas com Vírus da Imunodeficiência Humana, em situação ambulatorial ativa, com idade igual ou superior a 18 anos. As entrevistas foram realizadas a partir de questões que abordaram as necessidades de aprendizagem percebidas sobre saúde e autocuidado e barreiras e modalidades de aprendizagem preferidas de pacientes ambulatoriais com HIV/AIDS.	A maioria manifestou interesse pela saúde e autocuidado. Muitos identificaram vários tópicos como barreiras de aprendizagem e modalidades de aprendizagens preferidas.	IV

## DISCUSSÃO

Os estudos analisados nesta revisão apontaram a utilização do MPS pela enfermagem no cuidado a pessoas nos diferentes ciclos de vida e condições de saúde ^(20-27)^.

Na análise dos níveis das hierarquias das evidências ^(18)^, três estudos foram caracterizados como nível II ^(22,23,25)^, um como nível III ^(24)^, dois como nível IV ^(21,26)^ e dois como nível VI ^(20,27)^, demonstrando o desenvolvimento de estudos para gerar evidências científicas de alta qualidade e robustez. Esse fato é necessário e imprescindível para garantir a tomada de decisões pelos enfermeiros e a melhoria da assistência prestada.

No que tange à aplicabilidade das teorias de Nola Pender nos estudos analisados, observou-se que estas foram empregadas para formulação de intervenções em saúde ^(20,22-25)^, assim como na análise de comportamentos e percepções promotoras de saúde ^(21,26,27)^.

### Formulação de intervenções em saúde

Um dos estudos de intervenção analisados no presente estudo apontou que uma intervenção realizada por meio de visitas domiciliares para a realização de educação em saúde, baseada no MPS de Pender, favoreceu a identificação de comportamentos, da autoeficácia e de fatores pessoais (físicos, sociais e psicológicos) que impactam no engajamento do indivíduo com as ações de autocuidado. Verificou-se que a percepção positiva de autoeficácia da mãe, em relação à amamentação, esteve associada com o estabelecimento da amamentação (duração, esforço necessário para se dedicar e cuidar de seu bebê, pensamentos sobre a amamentação e capacidade de lidar com dificuldades) ^(23)^.

Nesse contexto, cabe destacar a necessidade das equipes atuantes na APS resgatarem continuamente os preceitos básicos da territorialização e utilizarem a visita domiciliar como oportunidade de conhecer as famílias cadastradas no território e identificar necessidades em saúde, a fim de direcionar ações estratégicas de promoção da saúde que atendam às diferentes realidades de cada território. Ademais, o fortalecimento do vínculo entre o enfermeiro e o usuário do serviço oportuniza ao profissional auxiliar no processo de reflexão sobre os hábitos de vida e comportamentos adotados, assim como na identificação de barreiras, vantagens e autoeficácia do indivíduo em relação à mudança de comportamentos que impactam a promoção da saúde, conforme o modelo em discussão.

Um ensaio clínico randomizado e controlado que teve como base o MPS utilizou, como recurso de intervenção, um site com conteúdos educacionais, vídeos e instrumentos que dispunham de um menu de perguntas para que as mães tirassem suas dúvidas em relação ao aleitamento.

Evidenciou-se que, no terceiro mês de acompanhamento, 88,6% dos bebês no grupo de intervenção estavam em aleitamento materno, enquanto que, no grupo controle, esse número foi de 33,3%. Em relação à introdução prematura de alimentos, o grupo de intervenção apresentou 11,4% dos bebês, comparado a 66,7% dos lactentes do grupo controle ^(22)^. Frente a isso, destaca-se, no contexto da APS, a relevância e a necessidade das ações de acompanhamento desempenhadas pelas equipes de Saúde da Família em relação à eficácia das ações de promoção da saúde.

Considerando a gestação e o puerpério, por exemplo, como uma fase do ciclo de vida de algumas mulheres, o vínculo estabelecido e as ações de cuidado e acompanhamento contínuos adotados pela equipe de saúde, no contexto da APS, podem favorecer o reconhecimento, por parte das mulheres, dos profissionais como componentes da rede de apoio social, tão necessária para a adoção de hábitos e comportamentos saudáveis.

Por sua vez, um estudo de intervenção composto por sessões de aconselhamento e desenvolvimento de habilidades comportamentais, baseado no MPS de Pender, apontou que o grupo experimental apresentou melhores pontuações em relação à responsabilidade com sua própria saúde, nutrição e relacionamento interpessoal ^(24)^. O fato de o MPS priorizar a autopercepção dos envolvidos sobre seus comportamentos, fomentar o desenvolvimento de novos hábitos e aprimorar os que já existem, pode colaborar para o engajamento das pessoas com sua própria saúde.

Embora sejam frequentes as discussões sobre a prevenção de complicações em pessoas com doença crônica, abordar ações de promoção da saúde (que incorporem o impacto das ações adotadas na qualidade de vida) também constitui um elemento necessário para a integralidade do cuidado no contexto da APS. Atinente a isso, um estudo que utilizou o MPS como base para uma intervenção realizada com pessoas com hipertensão apontou melhora no perfil clínico e na percepção de autoeficácia dos participantes desse grupo ^(25)^.

Além dos estudos de intervenção analisados, uma revisão integrativa descreveu um modelo teórico desenvolvido a partir da aplicação do conceito de envelhecimento saudável na diagramação do MPS de Pender, formando uma estrutura capaz de orientar a operacionalização do processo de enfermagem direcionado à promoção do envelhecimento saudável ^(20)^. Este resultado pode oferecer subsídios para a discussão das ações desenvolvidas na APS com enfoque na promoção da saúde da população idosa.

### Análise de comportamentos e percepções promotoras de saúde

A utilização do MPS para análise de comportamentos promotores de saúde em idosos ^(21)^ e para identificação da percepção sobre aprendizagem e autocuidado de pessoas convivendo com HIV^(26)^ elucidou a relevância da utilização do referido modelo para a compreensão das dimensões do comportamento promotor de saúde pelos profissionais, além da inclusão das percepções individuais para engajar o usuário nas ações de autocuidado. Embora a condição de saúde e o adoecimento sejam influenciados também pelos determinantes sociais, que nem sempre são passíveis de intervenção pelo próprio indivíduo, a utilização das premissas do MPS de Pender para pensar as ações de promoção da saúde possibilita a identificação de aspectos pessoais relacionados a crenças e motivação para mudança de comportamento, que podem refletir no autocuidado com a saúde.

As ações de promoção da saúde permitem a criação de um espaço onde é possível realizar ações de educação em saúde que possibilitem ao indivíduo tomar decisões conscientes (considerando a realidade de cada um) e refletir sobre seus comportamentos atuais. Tornando-se consciente dos hábitos e comportamentos, aumenta-se a chance de o indivíduo se engajar com sua condição de saúde e estar pronto para a mudança ^(27)^. Frente a isso, vale destacar que o modelo assistencial biomédico, centrado na doença e na prescrição medicamentosa, não responde à complexidade dos fatores influentes na condição de saúde e adoecimento da população, o que reitera a incorporação de tecnologias de cuidado centradas na pessoa e em suas particularidades.

Um estudo cujo objetivo foi analisar a abordagem da enfermagem baseada no MPS de Pender, no cuidado a pessoas com alteração no padrão de sono, identificou que a utilização de dispositivos eletrônicos constituiu a principal barreira para a mudança de comportamento, com vistas à promoção do sono. A partir desse resultado, foi proposta uma intervenção cujo objetivo foi reduzir a utilização de celular e televisão, utilizar técnicas de relaxamento e estabelecer um horário para dormir. Após a intervenção, verificou-se impacto positivo no padrão de sono dos participantes^(28)^.

Reitera-se a importância de ações desenvolvidas pelos profissionais de saúde que estimulem e apoiem o processo de autocuidado dos usuários e que incluam as necessidades e as preferências do próprio indivíduo. O autocuidado é uma prática realizada pela própria pessoa para si mesma, desenvolvida de forma autêntica, revisando valores e princípios por meio da autorreflexão, exibindo comportamentos de cuidado e considerando a integralidade das dimensões corporais (física, mental e espiritual), as quais são refletidas no ambiente em que vive ^(29)^. Destarte, de acordo com os preceitos do MPS, destaca-se a importância de o objetivo central da ação de cuidado planejada estar vinculado a um comportamento sobre o qual o indivíduo possa intervir, pois ao sentir-se confiante de que o objetivo é alcançável, aumenta-se a autoconfiança e a autoeficácia ^(9)^.

Mesmo sendo colocado no centro do cuidado e tendo suas preferências respeitadas, inicialmente os indivíduos podem ter dificuldade em estabelecer metas, e é nesse contexto que o enfermeiro atua como “apoiador” no processo de autoavaliação de seus comportamentos, de sua condição de saúde e na elaboração do plano de cuidados. Fornecer opções permite ao indivíduo escolher e aumentar o controle pessoal sobre o comportamento proposto.

### Limitações do Estudo

Cumpre assinalar que apenas um dos artigos analisados utilizou todas as categorias do MPS. Contudo, esse fator não exclui a validade dos resultados encontrados, uma vez que, de acordo com o modelo, as categorias podem ser trabalhadas separadamente, e dependendo do resultado que o autor deseja alcançar, não há necessidade de utilizar todas as categorias.

### Contribuições para a Área de Enfermagem e Saúde Pública

Os resultados do presente estudo elucidaram a aplicabilidade do MPS proposto por pender em diversos contextos. O planejamento de ações de promoção da saúde pelo enfermeiro na APS necessita considerar os aspectos biopsicossociais do indivíduo, a fim de favorecer seu engajamento no cuidado com sua própria saúde.

## CONCLUSÃO

As produções científicas da Enfermagem que utilizaram o MPS de Nola Pender na APS destacaram o impacto positivo na percepção de autoeficácia, reconhecimento de vantagens e barreiras em relação à mudança de comportamento, aumento do engajamento do indivíduo com sua condição de saúde e o papel do apoio social.

Contudo, cabe destacar que estes resultados não são suficientes para afirmar a efetividade de sua utilização no processo de trabalho do enfermeiro na APS, considerando a complexidade e multideterminação das condições de saúde e adoecimento. Isso porque os estudos mensuraram o impacto positivo da utilização do modelo na mudança de comportamento de formas variadas, incluindo questionários autoaplicáveis, entrevistas e indicadores de saúde específicos, o que pode limitar a comparabilidade dos resultados. Além disso, não há como desconsiderar a influência dos determinantes sociais da saúde nos hábitos e comportamentos dos indivíduos, fatores que necessitam de intervenções intersetoriais.
